# By the Way

**Published:** 1910-08-27

**Authors:** 


### By the Way.

### A PHYSICAL PECULIARITY OF WITCHES!

In "La Revue des Traditions Populaires" Dr. Desaivre reminds us that in the Middle Ages the question was gravely discussed as to whether Eve, and more particularly Adam, possessed navels. It cannot be shown that any definite conclusions were arrived at on the point, but in view of the fact that neither of the persons mentioned had lived an intra-uterine life, their possession of such a scar may be considered doubtful. On the other hand, at that time no such doubt would seem to have been entertained with regard to persons accused of sorcery: absence of umbilicus was considered a certain sign of supernatural or devilish birth, and even to this day the tradition is widespread that sorcerers have no navel. In mediæval times the miserable suspects were submitted to all kinds of hateful investigations to establish their guilt. The public scales were requisitioned to prove whether or not their weight corresponded with their seeming corpulency, or, as the saying was, whether they possessed the weight of good Christians. In addition experts and matrons, according to the sex of the accused, examined them for body-marks proving their satanic origin, absence of an umbilical depression being certainly considered an infallible sign. It is nevertheless a fact that such a condition can exist, and is to be explained by the imperfect reduction of an umbilical hernia, a by no means rare accident in the new-born. Almost every trace of the placental insertion may disappear, or at least be discoverable only after a minute examination. The author has seen such a case in a member of his own family who had been born prematurely. The peculiarity caused no inconvenience to its somewhat proud owner, whose astonishment was great when the author informed him that had he been born a few centuries earlier the condition of which he was so vain would have been sufficient to have caused him to be burnt alive!

### AN EIGHTEENTH-CENTURY MAISON-DE-SANTE.

An interesting sidelight is thrown upon the uses to which a private asylum could be put at the end of the reign of Louis XVI. and later under "The Terror," by an anecdote related by M. Lenôtre in Dr. Minime's "La Médecine Anecdotique," with reference to the *maison-de-santé* of Dr. Belhomme. In those times, as at the present day, it was by no means rare for a prisoner of rank to be declared irresponsible for his actions and sent to an asylum, whence, after a few months of genteel restraint, he was released. This was not unknown even during the Revolution, but as a rule the prisoner at that time preferred the hospitality of his asylum to the attentions of the King's procurators or of Fouquier-Tinville, and therefore remained where he was. In this way the Bastille and scaffold were cheated of many victims. The hero of M. Lenôtre's story was a certain Marquis de Saint P——, a young philosopher, who, discontented with a state of society in which, be it said, he was at liberty to indulge his every caprice, acclaimed the advent of an epoch of *equality and reason*, without foreseeing the rude shock which such a millennium had in store for such as he. One evening in 1787, on the arrival of Marie Antoinette at the opera, the Queen as usual made her three bows to the public amid much applause, which was, however, interrupted by a loud whistling noise proceeding from the pit. The guilty party, no other than our friend the Marquis, was immediately ejected into the corridor, taken to the police station, and thence removed to the Châtelet, where he was submitted to cross-examination. No trace of what subsequently happened to him can be found until 1837, when, according to the *Gazette des Tribuneaux*, an action was instituted against him at the Tribunal Civil de la Seine for *irreverence to Queen Marie Antionette*, an echo of a misdemeanour which had occurred fifty years previously. The history of what had happened to the prisoner during the intervening period is interesting. Placed in the asylum of Dr. Belhomme, to whom his family paid a large monthly pension, the Marquis devoted himself with assiduity to literary research, paying no attention to current events. Naturally the worthy doctor had no intention of dismissing such a well-paying patient, and even when the Revolution came and the patient's family was driven into exile and its estates forfeited, the nation continued to pay for the maintenance of one considered mentally defective. Then he remained during many changes of proprietorship, and there he would probably have remained even longer than 1837 had he not been desirous of publishing the fruit of his literary labours, a comparative study of the historians of the Greek decadence, which he proposed to dedicate *to His Majesty Louis XVI., King of France and Navarre, by his very devoted and obedient servant*. . . . It was only on the printer pointing out to him that it would be better to dedicate it to the "memory" of that monarch that the whole story came out. The Marquis knew nothing of the King's death, of the Revolution, of Napoleon, nor of the rest of the history of the troublous times through which the nation had passed. His trial was brought about on the initiative of the one remaining friend of his youth, as the result of which he was acquitted, the ban of lunacy removed, and his freedom given him. The asylum which had afforded him shelter all these years would seem to have been a comfortable place, with large, airy rooms and spacious gardens. An excellent cuisine contributed to its popularity, and the severity of its discipline was not such as to preclude the possibility of agreeable social entertainment and even of amorous adventures. Its proprietor, by the judicious blending of politics with medicine, was able to obtain for it a kind of tacit safe-conduct from the authorities, and, so long as their pensions were forthcoming, the prisoners which the latter sent him were safe from the attentions of Fouquier-Tinville. Opportunities for escape were not lacking, but it was rare that they were taken advantage of. M. Lenôtre had at first considerable difficulty in locating the whereabouts of the asylum, as no documents of the time appeared to give information on the point. One day, however, he chanced to be walking in the Rue de Charonne, when his gaze fell upon a high gateway in the style of Louis XVI., upon which rested a tablet clearly engraved with the words, *Maison de santé du Dr. Belhomme.* He found the establishment exactly in the same condition as it had been during the Revolution; next to it the former Hôtel Chabanais, which Belhomme had annexed when his "business" increased, the two houses surrounded still by the garden, broad and calm as a park, in which were dotted the old summer-houses built in the style of those of the Trianon. Alone of all the prisons of the Revolution does this one stand to-day intact, as though seeking absolution from posterity for having been the only refuge for pleasure and tender adventures during hours when all was blood and tears.

